# Temporary Nurse Staffing in the Context of Nursing Shortages: A Narrative Review of Workforce Flexibility, Organizational Trade-Offs, and Patient Safety

**DOI:** 10.3390/healthcare14193227

**Published:** 2026-10-01

**Authors:** Despoina Pappa, Anna Giga, Nikolaos Anastasiou, Vasiliki Georgousopoulou, Georgios Manomenidis

**Affiliations:** 1 Department of Nursing, Henry Dunant Hospital Center, 11526 Athens, Greece; 2 Nursing Specialty, 3rd Vocational Upper Secondary School, 10442 Athens, Greece; annagiga81@gmail.com; 3 Department of Medicine, Central Clinic of Athens, 10680 Athens, Greece; nikos.anastasiou@gmail.com; 4 Department of Nursing, Democritus University of Thrace, 68100 Alexandroupoli, Greece; vgeorgousopoulou@yahoo.gr (V.G.); gmanomen@nurs.duth.gr (G.M.)

**Keywords:** nursing agency, travel nurses, contract nurses, staffing adequacy, workforce planning, surge capacity, clinical integration, work environment, healthcare management

## Abstract

Nursing shortages require hospitals to balance staffing adequacy with flexibility in responding to vacancies, fluctuating demand, and unexpected service pressures. This focused narrative review examines temporary nurse staffing in acute hospitals, with primary emphasis on agency, travel, and contract registered nurses, and integrates evidence on workforce demand, operational flexibility, economic and workforce consequences, clinical integration, and patient safety. Evidence was identified through targeted PubMed and Scopus searches, citation tracking, and supplementary literature discovery. Temporary staffing can provide adaptive capacity by redistributing available nursing labor and reducing exposure to unfilled staffing requirements, but it does not eliminate underlying workforce scarcity. The evidence does not support treating temporary nurses as intrinsically less competent or inherently unsafe. Outcomes instead vary with the intensity and persistence of temporary staff use, baseline staffing adequacy, work environment, familiarity with local systems, orientation, and organizational integration. Limited supplementation may be operationally and economically useful in some contexts, whereas recurrent high reliance may increase coordination demands, workforce strain, and costs and may be associated with selected adverse outcomes. Temporary staffing is therefore best evaluated as one component of the overall staffing configuration and against realistic alternatives, including permanent staffing, overtime, flexible internal staffing, reduced capacity, or unresolved understaffing.

## 1. Introduction

Nurses constitute the largest occupational group in the global health workforce and are central to the delivery of safe, continuous, and high-quality care. Despite recent growth in the nursing workforce, substantial shortages and geographic inequalities persist. The World Health Organization (WHO) estimated approximately 29.8 million nurses worldwide in 2023 and a global shortage of about 5.8 million, with marked imbalance in workforce distribution across countries [1]. These aggregate shortages interact with workforce aging, retirement, recruitment difficulties, and retention pressures, but their operational consequences are experienced locally by healthcare organizations that must maintain safe staffing despite variable demand and incomplete permanent workforce capacity.

At the hospital level, staffing requirements fluctuate with vacancies, sickness absence, patient volume and acuity, seasonal pressures, specialty requirements, and unexpected service demands. Temporary nurse staffing provides one mechanism for increasing capacity when permanent personnel are unavailable or planned staffing is insufficient. Its use can reflect two overlapping organizational logics: deliberate flexibility, whereby temporary labor is used to accommodate short-term variation without maintaining permanent capacity for every possible peak, and labor scarcity, whereby temporary staff are required because substantive posts cannot readily be filled [2]. Agency, travel, and contract nurses may therefore function both as a planned flexible resource and as a response to constraints in the permanent nursing labor market.

Temporary staffing can also distribute available nursing labor across organizations and regions, providing surge capacity during geographically uneven demand shocks [3]. However, redistribution cannot resolve underlying workforce scarcity when demand pressures become widespread. Temporary staffing should therefore be understood as a mechanism for supplementing available capacity rather than as a substitute for long-term workforce supply, recruitment, and retention strategies.

The organizational implications of temporary staffing extend beyond headcount. Temporary personnel may help avoid unfilled staffing requirements and maintain service capacity, but their use may also involve higher visible labor costs, orientation and supervision requirements, and reduced familiarity with local systems, routines, escalation processes, and team relationships [4,5,6,7,8]. These issues should not be interpreted as evidence that temporary nurses are intrinsically less competent. Evidence comparing permanent and nonpermanent nurses has not demonstrated a consistent independent disadvantage attributable to temporary employment status itself, and observed differences in quality or outcomes are often intertwined with staffing adequacy and the work environment [8,9,10].

Patient-safety evidence is similarly heterogeneous. Some studies report associations between higher or more persistent temporary-staff use and selected adverse outcomes, whereas others find no independent relationship after adjustment or under conditions of more limited supplementation [9,10,11,12,13,14,15]. These findings are difficult to interpret in isolation because temporary staffing is often used under conditions of existing workforce pressure, creating substantial potential for confounding by indication. This issue, together with the importance of the realistic staffing counterfactual, is considered in greater detail in Section 4.2.

Previous reviews have addressed important but partly separate aspects of temporary nursing. Simpson and Simpson [16] and Birmingham et al. [17] examined agency nurses’ experiences and integration; Vander Weerdt et al. [10] synthesized evidence on travel nurses and patient outcomes; Telschow and Berg [7] examined induction and rapid orientation; Wolbers et al. [4] used a realist approach to explain how temporary-staffing outcomes depend on context, staffing adequacy, work environment, and access to situated knowledge; and Pani [18] considered management-related risks, benefits, leadership, and organizational support. Together, these reviews demonstrate that temporary staffing cannot be understood through employment status alone. They also leave value for a synthesis that connects why temporary staff are used with the operational, economic, workforce, integration, and patient-safety consequences of that use.

Accordingly, the contribution of the present review is not to determine whether temporary nurses are inherently beneficial or harmful, nor to propose a universal threshold for appropriate temporary staff use. Instead, it examines temporary nurse staffing as a workforce strategy and integrates evidence across labor-market demand, operational flexibility, economic and workforce consequences, clinical integration, and patient safety. Particular attention is given to the realistic staffing counterfactual, the intensity and persistence of temporary staff use, baseline staffing adequacy, and the familiarity and organizational integration of temporary personnel. Within this perspective, strategic supplementation and structural dependence are treated as interpretive points along a management continuum rather than as discrete or empirically validated staffing categories.

For the purposes of this review, temporary nurse staffing is used as an overarching term for RNs employed or deployed on a nonpermanent basis to supplement the substantive hospital workforce. The primary focus is on externally supplied agency, travel, and contract RNs. Evidence concerning internal hospital-bank, pool, supplemental, float, or other nonpermanent staffing arrangements is included only when it directly informs the review questions. Because these arrangements differ in employment relationship, organizational familiarity, and integration into clinical teams, findings from broader or mixed temporary-staffing categories are identified explicitly and are not interpreted as evidence specific to external agency nurses.

This narrative review therefore aims to critically examine temporary nurse staffing in the context of nursing shortages, with particular emphasis on agency, travel, and contract registered nurses (RNs), by integrating evidence on the drivers of temporary-staff demand, workforce flexibility and surge capacity, economic and workforce consequences, clinical integration, and patient safety. It further examines how staffing intensity, baseline staffing adequacy, the staffing counterfactual, familiarity with the clinical environment, and organizational integration shape the interpretation of temporary staffing within the broader hospital workforce configuration.

## 2. Materials and Methods

### 2.1. Review Design

This study was conducted as a focused narrative review examining temporary nurse staffing in acute-care hospital settings, with primary emphasis on externally supplied agency, travel, and contract RNs, within the broader context of nursing shortages and hospital workforce management. Evidence concerning internal hospital-bank, pool, supplemental, float, or other nonpermanent staffing arrangements was considered when it directly informed the review questions, but such evidence was not interpreted as specific to external agency staffing.

A narrative approach was selected because the relevant literature encompasses heterogeneous research questions, staffing definitions, study designs, levels of analysis, and outcomes, including labor-market demand, workforce flexibility, economic and workforce consequences, organizational and clinical integration, and patient safety. The purpose was therefore not to estimate a single pooled intervention or exposure effect, but to integrate complementary forms of evidence and examine how the implications of temporary staffing vary according to staffing context, intensity and persistence of use, baseline staffing adequacy, workforce familiarity, and organizational integration.

The development and reporting of this narrative review were informed by the Scale for the Assessment of Narrative Review Articles (SANRA), particularly its criteria relating to justification of the review, statement of aims, description of the literature search, referencing, scientific reasoning, and presentation of relevant evidence [19]. SANRA was used solely as a framework to inform the reporting quality and internal coherence of the review itself; it was not used to assess the methodological quality or risk of bias of individual primary studies. Primary studies were instead interpreted qualitatively according to relevant methodological characteristics, as described in Section 2.3, and no formal study-level risk-of-bias tool or numerical quality score was applied. A descriptive mapping of the SANRA criteria to the present review is provided in Supplementary Table S1.

### 2.2. Literature Search and Source Selection

The literature base was developed through targeted searches in PubMed and Scopus covering publications from 1 January 2000 through 30 August 2026. PubMed and Scopus were selected to provide complementary coverage across clinical nursing, patient-safety, workforce, management, and interdisciplinary literature, with citation tracking used to broaden source identification. Search concepts combined terms relating to temporary nurse staffing—including agency, temporary, travel, contract, supplemental, bank, pool, float, contingent, and other nonpermanent nursing arrangements—with terms relating to hospitals and acute care. These exposure concepts were combined with domain-specific terms concerning nursing shortages and staffing demand, workforce flexibility and surge capacity, economic and workforce consequences, orientation and clinical integration, work environment, patient safety, quality of care, and patient outcomes. Reference lists and cited literature from key empirical studies and relevant systematic, scoping, integrative, qualitative, realist, and narrative reviews were also examined to identify additional potentially relevant sources.

For the core empirical synthesis, inclusion criteria were: (1) peer-reviewed empirical studies; (2) studies conducted in acute-care hospital settings or studies directly relevant to hospital nursing labor markets and workforce arrangements; (3) studies in which external agency, travel, or contract RNs constituted a meaningful exposure or study population, or studies of hospital-bank, pool, float, supplemental, or mixed temporary staffing when these arrangements directly informed one or more review domains; and (4) studies reporting findings relevant to temporary-staff demand, workforce flexibility, economic or workforce consequences, clinical integration, work environment, patient safety, or quality of care. Review-level evidence, including systematic, scoping, integrative, qualitative, realist, and narrative reviews, was also included where relevant to contextualize the primary empirical literature and identify areas of convergence, inconsistency, and uncertainty. Exclusion criteria were: (1) studies in which temporary staffing was mentioned only incidentally; (2) studies of non-nursing agency labor that did not permit nurse-specific interpretation; and (3) evidence from settings outside acute hospital care without direct relevance to the review questions. Studies involving broader or mixed temporary-staffing categories were retained only when directly relevant, and their findings were not interpreted as evidence specific to external agency nurses. The search and selection process was designed to support a focused narrative synthesis rather than an exhaustive systematic review. Potentially relevant records identified through the database searches and citation tracking were evaluated for consistency with the review scope and the eligibility criteria described above. Titles and abstracts were initially examined for relevance, and full texts were reviewed when the temporary-staffing exposure, clinical setting, or relevance to the review domains could not be determined adequately from bibliographic information alone. Source selection for the core empirical synthesis was undertaken by two authors (D.P. and V.G.) through discussion and consensus. As this was a focused narrative review, formal independent duplicate screening and a PRISMA-based study-selection procedure were not undertaken. Database searching was conducted primarily using English-language search terms and focused primarily on English-language publications. No systematic search of non-English-language literature was undertaken; however, relevant non-English publications identified through supplementary citation tracking or review-level evidence were considered when sufficient information was available for reliable interpretation. The search structure and principal database-specific search concepts are summarized in Supplementary Table S2.

### 2.3. Narrative Synthesis Approach

The evidence was synthesized narratively because of substantial heterogeneity in staffing terminology, employment arrangements, exposure measurement, clinical setting, study design, and outcome definition. The synthesis was organized around five conceptual domains corresponding to the aims of the review: (1) nursing shortages and determinants of temporary-staff demand; (2) workforce flexibility and surge capacity; (3) economic and workforce consequences; (4) clinical integration, orientation, familiarity, and work environment; and (5) quality-of-care and patient-safety outcomes.

Particular attention was given to the definition of the temporary staffing exposure. Evidence concerning external agency, travel, or contract RNs was distinguished, where possible, from evidence concerning hospital-bank, pool, float, supplemental, or mixed temporary staffing. The synthesis also distinguished the presence of temporary staffing from its intensity, persistence, or proportion within the overall staffing configuration, because occasional supplementation and sustained high reliance may represent materially different organizational conditions. Staffing thresholds or breakpoints reported in individual studies were treated as study and setting-specific and were not interpreted as universal limits for appropriate or safe temporary-staff use.

Interpretive emphasis was informed by methodological characteristics rather than by a formal numerical quality score. Larger, multisite, longitudinal, and appropriately adjusted studies were given greater interpretive emphasis than smaller, single-center, ecological, or cross-sectional studies, while review-level evidence was used to assess consistency across individual findings and to clarify relevant contextual mechanisms. Attention was paid to potential confounding by overall staffing adequacy, patient acuity and case mix, workload, hospital and unit characteristics, practice environment, and temporal factors such as the COVID-19 pandemic. Because temporary staff are commonly deployed in response to existing staffing pressure, confounding by indication and potential reverse causation were considered explicitly when interpreting associations. Observational associations were not interpreted as demonstrating causal effects of temporary employment status.

No formal meta-analysis or study-level risk-of-bias scoring was undertaken. Instead, study design, exposure definition, sample and setting, statistical adjustment, potential confounding, temporal context, and the applicability of each staffing definition to the review question were incorporated into the interpretation of the evidence. Review-level evidence was used to contextualize and compare the primary empirical findings rather than to treat different temporary-staffing arrangements as directly interchangeable. The principal review-level evidence is summarized in Table 1, while detailed characteristics and findings of the primary empirical studies informing the synthesis are provided in Supplementary Table S3.

## 3. Results

### 3.1. Overview of Included Studies

The core empirical synthesis comprised 26 primary empirical studies, supplemented by seven review-level evidence syntheses summarized in Table 1. Seventeen of the primary studies were conducted in the United States, six in the United Kingdom, two in Switzerland, and one in Iran. The evidence base was methodologically heterogeneous and included cross-sectional and descriptive surveys, retrospective and longitudinal observational analyses, hospital- and unit-level panel or time-series studies, economic evaluation, observational communication research, and qualitative case-study evidence. Units of analysis ranged from individual nurses and hospital units to large multicenter patient cohorts and national labor-market data. Across the review domains, temporary staffing consistently emerged as a mechanism for responding to shortages, vacancies, and fluctuations in demand, whereas findings concerning economic consequences, workforce effects, organizational integration, and patient safety were more context dependent. Patient-safety evidence included both null or attenuated associations after adjustment and adverse associations under higher or more persistent temporary-staff exposure. Because several studies used broader or mixed temporary-staffing definitions, findings were interpreted according to the specific staffing exposure examined in each study. Detailed characteristics of the primary studies are provided in Supplementary Table S3.

### 3.2. Narrative Synthesis by Review Domain

Following the overview of included studies, the evidence is synthesized below by review domain. Throughout the synthesis, the terms agency, travel, or contract nurse are used only when the underlying evidence specifically concerns those externally supplied staffing arrangements. The broader term temporary staffing is retained when studies examine internal bank, pool, float, supplemental, or mixed nonpermanent staffing categories; findings from these broader exposures should not be interpreted as specific to external agency or travel nurses.

#### 3.2.1. Nursing Shortages and the Demand for Temporary Staffing

Temporary nurse staffing arises from a mismatch between required nursing capacity and the permanent workforce available at a given time. Although global nursing shortages provide an important background condition, hospital demand for temporary nurses is shaped more immediately by vacancies, sickness absence, patient volume and acuity, specialty requirements, and fluctuations in service demand [1,2,4]. Temporary staffing may therefore serve two overlapping purposes: providing planned flexibility when demand varies and compensating for labor scarcity when permanent positions cannot readily be filled.

Evidence supports both mechanisms. Seo and Spetz [2] found that demand for temporary agency RNs increased during periods of labor shortage but was also associated with inpatient activity, unexpected variation in demand, and employment costs. More recent studies extend this finding across different levels of analysis. Ahmadi Shad et al. [21] showed that temporary nurses were deployed preferentially when permanent RN availability was reduced or workload increased, although deployment did not eliminate all staffing deficits. At the labor-market level, Gottlieb and Zenilman [3] demonstrated substantial geographic responsiveness of travel-nurse supply to regional demand and compensation, while also showing that redistribution becomes more constrained when demand increases simultaneously across markets. Collectively, these findings indicate that temporary staffing can redistribute and supplement available nursing capacity but cannot create additional workforce supply without limit. Recurrent reliance may reflect sustained vacancies, retention problems, or inadequate substantive staffing capacity rather than successful flexibility alone [4]. Temporary staff use should therefore be interpreted within the workforce conditions that generate demand for it.

#### 3.2.2. Temporary Staffing as Adaptive Capacity: Flexibility and Surge Response

The principal operational value of temporary nursing is its capacity to adjust labor supply more rapidly than permanent recruitment generally allows. This flexibility can be useful for short-lived vacancies, seasonal variation, unexpected absence, fluctuating patient activity, and acute surges, particularly when maintaining permanent capacity for every possible peak would itself be inefficient [2,3,21]. The COVID-19 pandemic provided an unusually visible demonstration of this function, as travel nurses moved toward areas experiencing severe demand pressure [3].

Temporary staffing can therefore provide partial adaptive capacity rather than a complete substitute for substantive workforce capacity. Its practical contribution depends on both the staffing deficit it addresses and the environment in which temporary personnel are deployed. Realist evidence similarly suggests that access to local knowledge, collaboration, and organizational support can shape how effectively temporary nurses contribute [4].

#### 3.2.3. Economic and Workforce Trade-Offs

The economic implications of temporary staffing cannot be assessed adequately by comparing agency bill rates with permanent nurses’ hourly wages. Permanent employment includes benefits, leave, recruitment, and the cost of maintaining capacity during periods of lower demand, whereas temporary staffing incorporates different recruitment, administrative, and placement costs. The relevant economic question is therefore whether temporary staffing represents an efficient response to a particular staffing requirement relative to the realistic alternatives available [2,5,6].

Available evidence suggests that this relationship is conditional rather than uniformly favorable or unfavorable. Hurst and Smith [5] found greater costs in wards using temporary personnel, but those wards also carried greater workloads, limiting attribution of excess costs to temporary staff alone. Xue et al. [6] found that modest supplemental RN use could be economically efficient within a local staffing configuration, whereas heavier reliance was not. Saville et al. [22] provided a stronger counterfactual perspective: although eliminating understaffing was estimated to be cost-effective, achieving this through permanent staffing was more cost-effective and produced greater estimated health benefit than filling the same deficits through agency staff. Together, these studies suggest that temporary staffing can be economically rational as a source of marginal flexibility without implying that recurrent agency reliance is equivalent to sustained investment in substantive staffing.

Workforce consequences are likewise mixed. Greater exposure to temporary nurses has been associated with additional demands on permanent staff and lower organizational commitment through perceptions of illegitimate tasks [23], while qualitative evidence indicates that these burdens are strongly influenced by the familiarity of temporary staff with the clinical environment [24]. From the temporary-nurse perspective, travel nurses have reported greater turnover intention, physical fatigue, and perceived illegitimate tasks than staff nurses, but not uniformly poorer job satisfaction, emotional exhaustion, workload, or pay satisfaction [25]. Similarly, Fiore-Lopez et al. [26] found no meaningful overall difference between travel and nontravel nurses in perceptions of the work environment. The evidence therefore supports specific workforce trade-offs rather than a generalized claim that temporary employment produces poorer work experience.

At the management level, temporary staff expenditure should consequently be interpreted alongside vacancy rates, turnover, overtime, sickness absence, orientation requirements, service capacity, and patient outcomes. Persistent or increasing expenditure may be economically important not only because of agency costs themselves but because it can signal unresolved permanent-workforce problems.

#### 3.2.4. Clinical Integration, Situated Knowledge, and the Permanent Workforce

Many concerns associated with temporary staffing relate less to general professional competence than to local familiarity. An experienced RN entering an unfamiliar organization may lack knowledge of local electronic systems, medication processes, escalation pathways, equipment, routines, documentation practices, and informal communication networks [4,7,8]. These forms of situated knowledge are distinct from professional nursing competence and help explain why staffing numbers alone may not represent immediately usable clinical capacity.

Evidence on orientation reinforces this distinction. Telschow and Berg [7] found that temporary nurses require rapid access to practical local knowledge and appropriate sources of support, while orientation itself can impose additional demands on permanent staff. Travel-nurse studies similarly identify timely pre-assignment information, day-one system access, unit-specific operational information, and competency clarification as important onboarding requirements [27,28]. Orientation should therefore be understood as enabling rapid local functionality rather than remedying presumed deficiencies in professional capability.

The importance of familiarity extends beyond formal orientation. Wolbers et al. [4] described situated knowledge as a mechanism through which temporary-staffing models may influence continuity, teamwork, and permanent-staff workload. When unfamiliar temporary nurses are introduced into already strained environments, substantive staff may need to provide greater supervision, coordination, and patient-specific knowledge; when organizational support and collaboration are stronger, temporary personnel may contribute more effectively. Observational communication evidence is consistent with this interpretation: differences associated with staff consistency suggest that continuity of presence can shape the way clinical information is exchanged, without demonstrating that temporary nurses communicate less effectively [29].

Consequently, two units with the same proportion of temporary nursing hours may have materially different staffing configurations if one repeatedly deploys familiar personnel with established access and the other relies on frequently changing staff with limited local preparation. Repeat placement, unit familiarity, orientation, and integration may therefore represent important dimensions of temporary-staff exposure that are not captured by simple percentages of hours or shifts.

Local access, familiarity, and integration should therefore be considered relevant contextual dimensions of temporary staff deployment when interpreting patient-safety outcomes [4,7,15].

#### 3.2.5. Patient Safety and Quality: Dose, Context, and Confounding

Evidence concerning temporary staffing and patient safety is heterogeneous. Studies differ in whether they examine agency, travel, contract, hospital-bank, supplemental, or mixed temporary staff; whether exposure is measured as presence, hours, proportions, or categorical thresholds; and whether analyses account adequately for staffing levels, case mix, work environment, and temporal conditions [4,8,9,10]. Detailed characteristics of the primary studies are provided in Supplementary Table S3, while the principal review-level evidence is summarized in Table 1. A compact comparison of the principal quantitative studies informing the patient-safety synthesis is provided in Table 2.

One group of studies provides evidence against an independent, uniformly adverse effect of temporary employment status. Associations between supplemental-staff use and poorer care have been attenuated after accounting for staffing adequacy and the practice environment [8,9], and Xue et al. [30] found no significant relationship between agency-employed supplemental RNs and a range of patient outcomes. Infection-specific evidence is similarly inconsistent: temporary staffing was not independently associated with CLABSI or VAP in the ICU analysis by Bae et al. [31], whereas perceived staffing and resource adequacy showed stronger relationships with infection outcomes.

At the same time, several studies suggest that staffing intensity and composition may matter. Higher levels of temporary staffing have been associated with selected falls, pressure injuries, care left undone, and mortality outcomes [11,12,32,33,34]. These findings should not be combined to infer a single dose–response curve because the staffing definitions, populations, outcomes, and study-specific exposure categories differ substantially. In particular, Dall’Ora et al. [12] combined hospital-bank and agency nurses, illustrating why findings from broader temporary categories cannot be attributed specifically to external agency staffing.

Recent pandemic-era studies reinforce the absence of a uniform outcome pattern. Polancich et al. [35] reported an association between travel-RN volume and length of stay but not consistent associations with several nursing-sensitive outcomes, while Taylor et al. [36] found relationships between agency staffing and selected pediatric intensive-care outcomes that varied by year and endpoint. These studies were conducted under exceptional workforce and patient-demand conditions and therefore carry substantial potential for temporal and organizational confounding. The broader problem of confounding by indication is discussed in Section 4.2.

The staffing counterfactual is particularly important. Griffiths et al. [13] found that low RN staffing was associated with increased mortality, and that greater temporary-staff exposure also carried a modest adverse association; however, model-based analyses suggested that using temporary staff to address staffing deficits was preferable to leaving those deficits unfilled. Similarly, Pittman et al. [14] and Bartock et al. [15] identified associations between higher agency use and selected safety outcomes, but these were neither universal across outcomes nor sufficient to establish a causal effect of agency nurses themselves. Study-specific breakpoints should therefore be interpreted as signals for further scrutiny rather than as universal limits for safe staffing.

Review-level syntheses similarly report heterogeneous findings and emphasize the importance of staffing adequacy, familiarity, work environment, and organizational support in interpreting temporary-staffing outcomes [4,10,18].

Taken together, the heterogeneous evidence does not support characterizing temporary nurses generally, or external agency and travel nurses specifically, as inherently unsafe. Findings derived from broader or mixed temporary-staffing exposures should not be attributed specifically to externally supplied nurses. Patient-safety findings are more appropriately interpreted within the overall staffing configuration, including baseline staffing adequacy, intensity and persistence of temporary-staff use, patient complexity, familiarity with the clinical environment, and organizational integration.

## 4. Discussion

### 4.1. From Strategic Supplementation to Structural Dependence

The principal contribution of this review is the integration of evidence that has often been considered separately across labor-market, operational, economic, workforce, clinical-integration, and patient-safety perspectives. This cross-domain view suggests that the same temporary-staffing mechanism may have materially different implications depending on whether it is used to accommodate short-lived variation in demand or repeatedly to compensate for insufficient substantive workforce capacity.

Within this interpretation, strategic supplementation and structural dependence represent positions along a management continuum rather than empirically validated staffing categories. Limited or episodic supplementation may provide useful flexibility within an otherwise sustainable staffing model, whereas recurrent high-intensity use may accompany unresolved vacancies, retention problems, or persistent misalignment between substantive staffing capacity and service demand. No universal proportion of temporary staffing can currently define where one condition becomes the other.

The staffing counterfactual is central to this distinction. Temporary staffing may be inferior to an available and appropriately deployed permanent RN while still being preferable to an unfilled staffing requirement. Griffiths et al. [13] showed that model-based use of temporary RNs to address low staffing reduced the estimated mortality risk relative to leaving deficits unfilled, although it did not reproduce the configuration associated with adequate substantive staffing. At a broader workforce level, Gottlieb and Zenilman [3] demonstrated the capacity of travel-nurse markets to redistribute labor during demand shocks, whereas Saville et al. [22] estimated greater cost-effectiveness and health benefit when understaffing could instead be eliminated through permanent staffing. These findings illustrate why temporary staffing cannot be evaluated independently of the alternative realistically available.

Accordingly, the practical question is whether temporary staff use remains proportionate to the staffing problem it is intended to address. Figure 1 summarizes the principal dimensions relevant to this interpretation, including staffing demand, the realistic counterfactual, intensity and persistence of use, and organizational integration. These dimensions constitute an interpretive management framework rather than a validated classification or causal model.

### 4.2. Interpreting Heterogeneous Evidence

Several features of the underlying literature explain why apparently conflicting findings can coexist. First, the label temporary nurse encompasses organizationally different arrangements, including external agency and travel nurses, internal hospital-bank personnel, supplemental RNs, contract nurses, and float staff. These categories differ in employment relationship, continuity of placement, familiarity with local systems, and integration within clinical teams [4,10]. Treating them as interchangeable can obscure clinically relevant differences in how temporary staffing functions.

Second, exposure has been measured inconsistently. Studies variously examine the presence of temporary staff, hours or proportions of temporary staffing, study-specific thresholds, or categories of high and low reliance. The available evidence suggests that intensity may matter, but differences among exposure measures, populations, and outcomes preclude derivation of a universal safe or unsafe percentage [11,12,13,14,15].

Third, temporary staffing is particularly vulnerable to confounding by indication. Hospitals and units generally increase temporary staff use because substantive staffing is already insufficient, demand has risen, sickness absence is high, recruitment is difficult, or patient complexity has changed. These same circumstances may independently influence outcomes. Consequently, associations between agency use and adverse events may reflect effects of the temporary-staffing configuration, the organizational conditions that generated temporary-staff demand, or both. This problem is especially important in studies conducted during the COVID-19 period, when staffing disruption, acuity, demand, and temporary-staff use changed simultaneously [14,15,35,36].

Most available studies are observational, and even stronger longitudinal analyses cannot eliminate residual confounding. The evidence therefore supports conditional rather than causal conclusions. It is not sufficient to infer that temporary nurses caused an adverse outcome because higher agency exposure and poorer outcomes occurred together; equally, null associations in one staffing context should not be generalized to all levels or patterns of temporary staff use.

### 4.3. Implications for Workforce Governance and Clinical Practice

The following implications should be interpreted as evidence-informed considerations for workforce governance and clinical practice rather than as causal recommendations or prescriptive staffing thresholds. An evidence-informed approach is to monitor temporary staffing as part of the overall staffing configuration rather than solely as an expenditure category. Where feasible, workforce monitoring may distinguish between substantive RN staffing, internal flexible staffing, external agency or travel hours, overtime, vacancies, sickness absence, and measures of overall staffing adequacy. Recurrent increases in temporary staff use should prompt examination of whether they reflect genuine short-term variation or unresolved recruitment, retention, workload, or baseline-capacity problems [13,14].

Orientation and integration should likewise be considered components of temporary staff deployment rather than peripheral administrative tasks. Evidence supports timely pre-assignment information, access to necessary electronic systems and local protocols, unit-specific information, clarification of competencies and escalation pathways, and readily identifiable clinical support [7,27,28]. Repeated placement of temporary nurses in familiar environments may also reduce the amount of situated knowledge that has to be reconstructed at each deployment [4,24,29]. However, current evidence does not establish a universally optimal orientation format or duration.

This issue is relevant not only to temporary nurses but also to the substantive workforce. Numerical increases in staffing may yield less than equivalent increases in immediately usable clinical capacity when permanent staff must simultaneously provide extensive orientation, supervision, problem solving, or local knowledge. This should not be interpreted as evidence of deficient professional competence among temporary nurses; it reflects the organizational cost of unfamiliarity [4,23,28]. Workforce planning should therefore consider continuity, familiarity, team composition, and the support capacity of permanent staff alongside total RN hours.

Policy responses should consequently avoid treating temporary staffing as either inherently undesirable or fully interchangeable with substantive staffing. Removing access to temporary staff may leave clinically important deficits unfilled, whereas repeated high reliance may conceal or perpetuate underlying workforce instability. The available evidence is more consistent with an approach that prioritizes adequate substantive staffing capacity while retaining temporary staffing as a flexible option whose use, intensity, and organizational integration are monitored in relation to local workforce conditions.

### 4.4. Strengths, Limitations, and Research Priorities

A strength of this review is its integration of labor-market, operational, economic, workforce, clinical-integration, and patient-safety evidence within a single management-oriented perspective. Several limitations should nevertheless be acknowledged. As a focused narrative review, the search was designed to identify relevant evidence rather than achieve systematic completeness; no PRISMA-based study-selection process, formal risk-of-bias tool, or meta-analysis was applied. The search was limited to PubMed and Scopus and did not include CINAHL; consequently, some nursing-specific workforce, management, or administration literature indexed primarily in CINAHL may not have been identified. This limitation should be considered in light of the focused narrative, rather than systematic, design of the review. Similarly, because database searching was conducted primarily in English, relevant non-English-language evidence may have been underrepresented. The underlying literature is also limited by inconsistent terminology and exposure definitions, incomplete separation of external agency or travel nurses from internal flexible staffing, concentration of evidence in the United States and United Kingdom, and the inclusion of several pandemic-era studies conducted under exceptional workforce conditions. These features limit causal interpretation and direct transferability across healthcare systems.

Future studies could also operationalize the proposed continuum by longitudinally tracking unit- or hospital-level temporary-staff use alongside vacancy rates, turnover, sickness absence, unfilled staffing requirements, and the recurrence or persistence of temporary-staff deployment. Examining whether sustained increases in temporary staffing co-occur with persistent workforce instability after accounting for baseline staffing adequacy and service demand could provide an empirical basis for distinguishing strategic supplementation from structural dependence.

More broadly, future research should distinguish external agency and travel nurses from internal flexible staffing and characterize temporary staffing by duration, repeat placement, prior unit experience, orientation, and familiarity with local systems. Patient-level longitudinal and quasi-experimental designs are needed to address confounding by indication and explicitly compare realistic staffing counterfactuals, including permanent staffing, overtime, internal bank use, reduced capacity, or unresolved understaffing. Economic and workforce outcomes should be evaluated alongside patient outcomes so that temporary staffing can be assessed as a system-level workforce strategy rather than solely as an employment category.

## 5. Conclusions

Temporary nurse staffing can provide adaptive capacity when hospitals face vacancies, fluctuating demand, or short-term staffing deficits, but its implications depend on the broader staffing configuration rather than temporary employment status alone. Baseline staffing adequacy, the intensity and persistence of temporary staff use, the staffing alternative realistically available, and the familiarity and integration of temporary personnel all shape how temporary staffing functions in practice.

Temporary staffing is therefore best interpreted along an interpretive continuum from strategic supplementation to structural dependence. Limited and appropriately integrated use may help maintain service capacity when the alternative is an unfilled staffing requirement, whereas recurrent high-intensity reliance may signal unresolved workforce instability and may be accompanied by higher costs, coordination demands, reduced continuity, or selected adverse outcomes. No universal threshold can currently define when temporary staffing becomes inappropriate or unsafe. The central policy question is not whether temporary staffing should be eliminated, but how, when, and to what extent it can provide flexible capacity without becoming a recurrent substitute for a sustainable substantive nursing workforce.

## Figures and Tables

**Figure 1:**
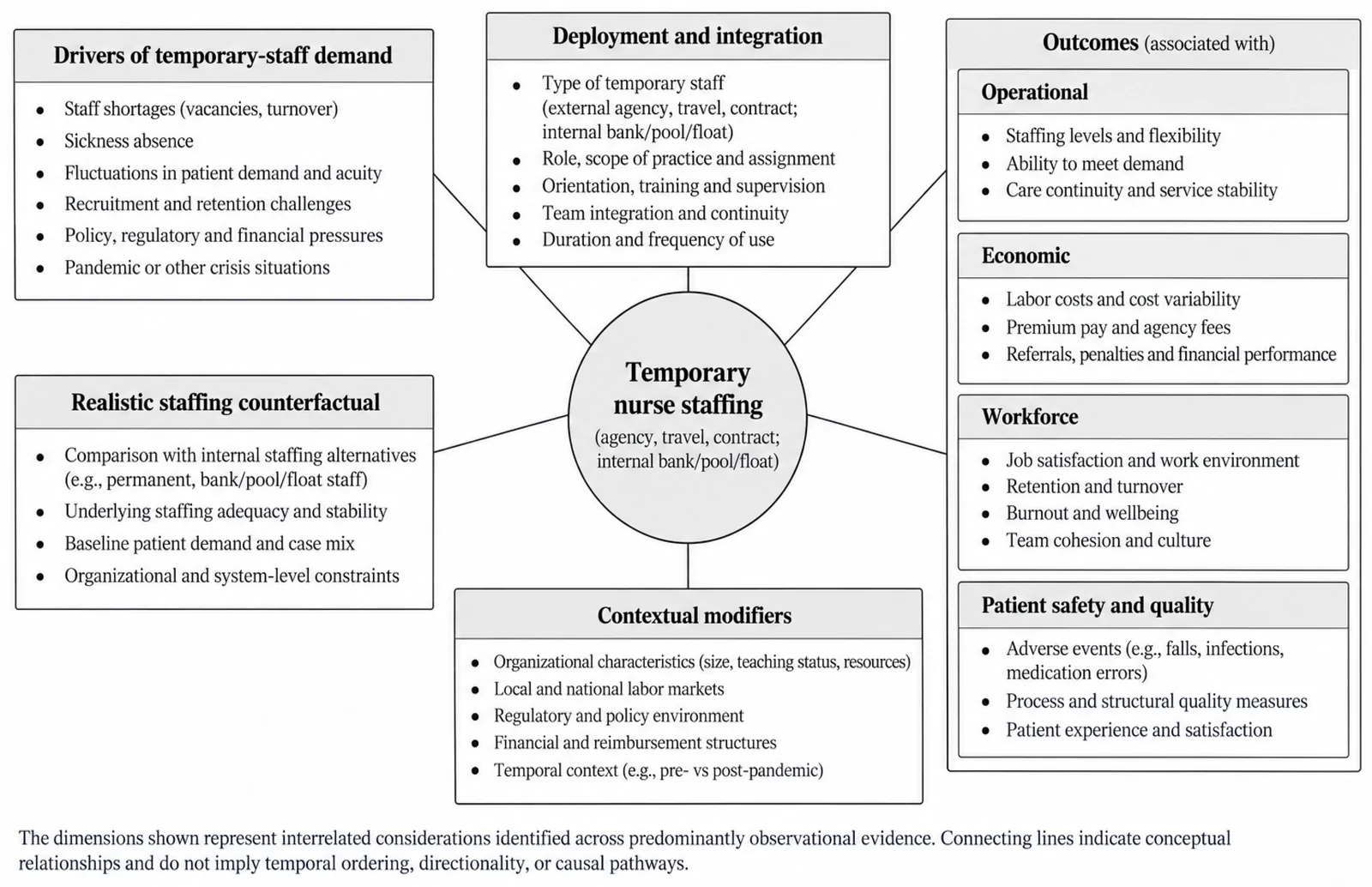
Non-directional interpretive framework for evaluating temporary nurse staffing within the overall hospital workforce configuration.

**Table 1:** Key review-level evidence on temporary, agency, and travel nurse staffing.

Review	Scope	Evidence Base	Main Contribution	Limitation Relevant to the Present Review
Simpson and Simpson [16]	Experiences and characteristics of agency nurses	8 international studies	Agency nurses valued flexibility and autonomy but reported professional isolation, difficulties with permanent staff, and fewer training and career-development opportunities.	Focused primarily on agency nurses’ lived experiences rather than hospital staffing intensity, patient outcomes, or economic consequences; settings were not restricted exclusively to acute hospitals.
Birmingham et al. [17]	Experiences of agency RNs and their integration into clinical practice	2 primary qualitative studies	Identified four central themes: orientation, allocation of agency nurses, professional isolation, and limited access to education and professional development.	Extremely small primary evidence base; focused on nurses’ experiences rather than organizational or patient-level effects.
Hermes and Petersen-Ewert [20]	Agency work in ICU/IMC settings	3 studies	Evidence was insufficient to demonstrate a consistent independent effect of agency work on patient outcomes; qualifications and working conditions appeared important to interpretation.	Very small evidence base and narrow ICU/IMC focus.
Vander Weerdt et al. [10]	Travel nurses and patient outcomes	21 articles	Found heterogeneous and mixed findings; staffing levels, work environment, exposure definitions, and study design substantially affected interpretation of associations with patient outcomes.	Focused predominantly on patient outcomes rather than temporary staff demand, economics, permanent-workforce consequences, or organizational integration.
Telschow and Berg [7]	Induction and rapid orientation of temporary nurses in hospitals	8 qualitative studies	Identified the importance of rapid orientation, local knowledge, designated support, and collaboration; induction also imposed time and coordination demands on permanent staff.	Used a broader definition of temporary nursing that included agency nurses as well as hospital-employed pool/float staff working in unfamiliar units.
Pani [18]	Risks, benefits, and management of temporary nurses	Narrative literature review; no formal study count reported in the abstract	Emphasized that many difficulties attributed to temporary staffing may reflect inconsistent leadership, inadequate induction, and limited organizational support; temporary nurses may provide flexibility and expertise when effectively supported.	Narrative management-focused review without a formal effect-estimation approach or clearly reported quantitative evidence base.
Wolbers et al. [4]	Temporary-nurse staffing models and context-mechanism-outcome relationships	49 studies	Heavy reliance on unfamiliar temporary nurses could threaten continuity and burden permanent staff, particularly when baseline staffing was low; supportive environments, collaboration, situated knowledge, onboarding, and orientation could facilitate positive contributions.	Uses a broader definition of temporary staffing than external agency nursing and applies realist rather than comparative effect-estimation methodology.

Note: Abbreviations: RN, registered nurse; ICU, intensive care unit; IMC, intermediate care unit. Review-level evidence was used to contextualize and interpret the primary empirical literature rather than to treat findings from different temporary-staffing models as directly interchangeable. Differences in employment arrangements, organizational familiarity, review methodology, and underlying staffing contexts limit direct comparison across reviews.

**Table 2:** Selected primary quantitative evidence informing the patient-safety synthesis.

Study	Temporary-Staffing Exposure	Principal Outcome(s)	Direction of Reported Association
Aiken et al. [9]	Agency-employed supplemental RNs	Mortality; failure to rescue	No independent association after adjustment for staffing and work environment.
Bae et al. [11]	External temporary RN hours	Falls; medication errors; nurse back injuries	>15% external temporary RN hours was associated with more reported falls and nurse back injuries; patterns for medication errors differed at moderate exposure.
Xue et al. [30]	Agency-employed supplemental RNs	Mortality; medication errors; falls; pressure ulcers; patient satisfaction	No significant associations with the outcomes examined.
Bae et al. [31]	Temporary nursing staff	CLABSI; VAP	No significant association with CLABSI or VAP; staffing/resource adequacy showed stronger associations with infection outcomes.
Bae et al. [32]	Contract/temporary RN hours	Falls; injury falls; pressure ulcers	Higher temporary RN staffing was associated with higher fall and injury-fall rates; no association with pressure ulcers.
Dall’Ora et al. [12]	Combined hospital-bank and agency RN staffing	Mortality	Modest temporary RN use was not associated with increased mortality; high exposure was associated with higher mortality hazard.
Ferguson et al. [33]	Contract/travel nurses	Hospital-acquired pressure injuries; falls	Highest traveler proportions were associated with greater pressure-injury prevalence/incidence; no consistent association with falls.
Senek et al. [34]	Proportion of temporary nursing staff on shifts	Care left undone	Higher temporary-staff proportions were associated with greater odds of reported care left undone.
Polancich et al. [35]	Travel RN FTEs	Length of stay; nursing-sensitive outcomes	Travel RN FTEs correlated positively with length of stay; several infection and fall outcomes were not significantly associated.
Griffiths et al. [13]	Agency and hospital-bank temporary RNs analyzed separately	Mortality; staffing substitution	Temporary staffing partly mitigated mortality risk associated with low staffing but did not reproduce the configuration associated with adequate substantive RN staffing.
Taylor et al. [36]	Annual agency-nurse proportion	VAP; CLABSI; PICU complications	Associations varied by year and outcome; selected adverse associations occurred in 2020–2021 but were not consistent across years or outcomes.
Pittman et al. [14]	Agency nurse hours	Patient-safety indicators	Agency hours were associated with selected outcomes, particularly pressure ulcers and perioperative hemorrhage/hematoma; associations were not universal across the 10 indicators.
Bartock et al. [15]	Agency/travel RN shifts	Patient-safety indicators; infections	Agency staffing > 10% was associated with higher total PSI burden; adjusted infection outcomes were explained more strongly by case mix than by agency-staffing tier.

Abbreviations: RN, registered nurse; CLABSI, central line-associated bloodstream infection; VAP, ventilator-associated pneumonia; FTE, full-time equivalent; PICU, pediatric intensive care unit; PSI, patient safety indicator. Note: Exposure definitions differ across studies; findings involving hospital-bank, supplemental, mixed, or other temporary-staffing categories should not be interpreted as evidence specific to external agency nurses. Study-specific thresholds are not universal safe or unsafe staffing limits.

## Data Availability

No new data were created or analyzed in this study. Data sharing is not applicable to this article.

## Supplementary Materials

The following supporting information can be downloaded at: https://www.mdpi.com/article/10.3390/healthcare14193227/s1. Table S1: SANRA-Informed Reporting Map for the Narrative Review; Table S2: Literature Search Strategy Used to Support the Focused Narrative Review; Table S3: Primary Empirical Studies Informing the Narrative Review of Temporary Nurse Staffing.

## References

[1] World Health Organization (2025). State of the World’s Nursing 2025: Investing in Education, Jobs, Leadership and Service Delivery.

[2] Seo S., Spetz J. (2013). Demand for temporary agency nurses and nursing shortages. Inquiry.

[3] Gottlieb J.D., Zenilman A. (2026). When Nurses Travel: Labor Supply Responses to Peak Demand for Nurses. Rev. Econ. Stat..

[4] Wolbers I., van Oostveen C., Stalpers D., Cummings G.G., Tate K., Schoonhoven L., Lalleman P., ResoNANCE Consortium (2026). Nurse staffing models that rely on employment of temporary nurses: A realist review. Int. J. Nurs. Stud. Adv..

[5] Hurst K., Smith A. (2011). Temporary nursing staff—Cost and quality issues. J. Adv. Nurs..

[6] Xue Y., Chappel A.R., Freund D.A., Aiken L.H., Noyes K. (2015). Cost outcomes of supplemental nurse staffing in a large medical center. J. Nurs. Care Qual..

[7] Telschow J., Berg A. (2025). Induction and rapid orientation of agency nursing staff in the hospital setting: A systematic synthesis of qualitative studies. J. Adv. Nurs..

[8] Aiken L.H., Xue Y., Clarke S.P., Sloane D.M. (2007). Supplemental nurse staffing in hospitals and quality of care. J. Nurs. Adm..

[9] Aiken L.H., Shang J., Xue Y., Sloane D.M. (2013). Hospital use of agency-employed supplemental nurses and patient mortality and failure to rescue. Health Serv. Res..

[10] Vander Weerdt C., Peck J.A., Porter T. (2023). Travel nurses and patient outcomes: A systematic review. Health Care Manag. Rev..

[11] Bae S.-H., Mark B.A., Fried B. (2010). Use of temporary nurses and nurse and patient safety outcomes in acute care hospital units. Health Care Manag. Rev..

[12] Dall’Ora C., Maruotti A., Griffiths P. (2020). Temporary staffing and patient death in acute care hospitals: A retrospective longitudinal study. J. Nurs. Scholarsh..

[13] Griffiths P., Saville C., Ball J., Culliford D., Jones J., Lambert F., Meredith P., Rubbo B., Turner L., Dall’ora C. (2024). Nursing team composition and mortality following acute hospital admission. JAMA Netw. Open.

[14] Pittman P., Tiunn H.-L., Luo Q., Herron M., Tatum D., Martin J. (2025). Increased utilization of overtime and agency nurses and patient safety. JAMA Netw. Open.

[15] Bartock J., Green A., Nahra R., Bordogna A., Davoodi A., Rachoin J.-S. (2026). Association of travel nursing with quality outcomes in hospitalized patients. Am. J. Manag. Care.

[16] Simpson K., Simpson R. (2019). What do we know about our agency nurse population? A scoping review. Nurs. Forum.

[17] Birmingham C., van de Mortel T., Needham J., Latimer S. (2019). The experiences of the agency registered nurse: An integrative literature review. J. Nurs. Manag..

[18] Pani C. (2026). From strain to strength: Enhancing the benefits of employing temporary nurses. Nurs. Manag..

[19] Baethge C., Goldbeck-Wood S., Mertens S. (2019). SANRA—A scale for the quality assessment of narrative review articles. Res. Integr. Peer Rev..

[20] Hermes C., Petersen-Ewert C. (2022). Leih- und Zeitarbeit in der Intensivpflege: Auswirkungen auf die Patientenversorgung der Überwachungs- und Intensivstationen [Agency work in intensive care: Impact of temporary contract work on patient care in intermediate care and intensive care units]. Med. Klin. Intensivmed. Notfmed..

[21] Ahmadi Shad M., Khorasanizadeh M., Musy S.N., Zúñiga F., Atoof F., Simon M. (2025). Temporary nurse deployments: A time-series analysis of shift scheduling dynamics and staffing level alignment. Int. J. Nurs. Stud. Adv..

[22] Saville C., Jones J., Meredith P., Dall’Ora C., Griffiths P. (2025). Cost-effectiveness of eliminating hospital understaffing by nursing staff: A retrospective longitudinal study and economic evaluation. BMJ Qual. Saf..

[23] Gahrmann C., Klumb P.L. (2024). Investigating the impact of temporary nurses on permanent nurses’ commitment via perceptions of illegitimate tasks: A cross-sectional study. J. Adv. Nurs..

[24] Bajorek Z., Guest D. (2019). The impact of temporary staff on permanent staff in accident and emergency departments. J. Organ. Eff. People Perform..

[25] Spector P.E., Pindek S., Hayman M.R., Howard D.J., Arvan M.L. (2024). Travel nurse work experiences: A comparison of staff and travel nurses’ burnout and job attitudes. Nurs. Manag..

[26] Fiore-Lopez N., Raso R., Downs S., Voss J., Kollman S., Clark A., Farber B., Gerlick J., Schneider B., Jones N. (2025). A national study of travel and nontravel nurses’ perceptions of the work environment. J. Nurs. Adm..

[27] Bethel C., Olson S., Bay C., Uyeda T., Johnson K. (2019). Travel nurse onboarding: Current trends and identified needs. J. Nurs. Adm..

[28] Tuttas C.A. (2015). Job integration factors as predictors of travel nurse job performance: A mixed-methods study. J. Nurs. Care Qual..

[29] Schmelzer S.D., Berger S., Seelandt J.C., Erden Z., Liberatore F. (2025). Impact of temporary nursing staff on communication patterns: An observation study during daily nurse huddles. BMJ Open Qual..

[30] Xue Y., Aiken L.H., Freund D.A., Noyes K. (2012). Quality outcomes of hospital supplemental nurse staffing. J. Nurs. Adm..

[31] Bae S.-H., Brewer C.S., Kelly M., Spencer A. (2015). Use of temporary nursing staff and nosocomial infections in intensive care units. J. Clin. Nurs..

[32] Bae S.-H., Kelly M., Brewer C.S., Spencer A. (2014). Analysis of nurse staffing and patient outcomes using comprehensive nurse staffing characteristics in acute care nursing units. J. Nurs. Care Qual..

[33] Ferguson A., Bradywood A., Williams B., Blackmore C.C. (2020). Association of use of contract nurses with hospitalized patient pressure injuries and falls. J. Nurs. Scholarsh..

[34] Senek M., Robertson S., Ryan T., King R., Wood E., Tod A. (2020). The association between care left undone and temporary nursing staff ratios in acute settings: A cross-sectional survey of registered nurses. BMC Health Serv. Res..

[35] Polancich S., Miltner R., Montgomery A., Dick T., Poe T., Brown D., Patrician P.A. (2023). Quality matters: Examining the impact of nurse staffing challenges on clinical outcomes. J. Healthc. Manag..

[36] Taylor W.M., Pelletier J., Heneghan J.A., Ramgopal S., Toth K.M., Prout A., Woeltje M., Butler G., Fabio A., Evans I. (2025). Pediatric intensive care nurse staffing measures and patient outcomes during the COVID-19 pandemic. JAMA Netw. Open.

