# Supplementary material for: Temporary Nurse Staffing in the Context of Nursing Shortages: A Narrative Review of Workforce Flexibility, Organizational Trade-Offs, and Patient Safety
Source: Healthcare (Basel). 2026 Oct 1;14(19):3227. doi: 10.3390/healthcare14193227 (PMC13665057; doi:10.3390/healthcare14193227)
Supplement: Supplementary file 1 [file healthcare-14-03227-s001.zip › healthcare-4579420-supplementary.pdf]

Supplementary Table S1. SANRA-Informed Reporting Map for the Narrative Review

SANRA criterion	How addressed in the present review	Manuscript location
**Justification of the article's importance**	The Introduction establishes the continuing nursing shortage, the operational need for flexible staffing, and the organizational and patient-safety relevance of temporary nurse staffing.	Introduction, paragraphs 1–5
**Statement of concrete aims**	The review aim specifies the focus on temporary nurse staffing, particularly agency, travel, and contract RNs, and identifies the principal domains of synthesis.	End of Introduction
**Description of the literature search**	The Methods describe the databases and supplementary discovery approaches used, publication coverage, search concepts, source-selection principles, and the narrative rather than systematic nature of the review. Detailed search information is provided in Supplementary Table S2.	Section 2.2; Supplementary Table S2
**Referencing**	Empirical and review-level claims are supported by primary studies and relevant evidence syntheses. Broader temporary-staffing categories are distinguished from agency-specific evidence where possible.	Throughout manuscript; Table 1; Supplementary Table S3
**Scientific reasoning**	Evidence is interpreted with attention to exposure definition, staffing intensity, staffing counterfactuals, confounding by indication, work environment, familiarity, and the observational nature of much of the literature.	Sections 2.3, 3.1–3.5, and 4.2
**Appropriate presentation of relevant evidence**	Review-level evidence is summarized in Table 1, primary empirical studies in Supplementary Table S2, and the cross-domain interpretation in Figure 1. Study-specific thresholds are not presented as universal limits.	Table 1; Supplementary Table S3; Figure 1

Note: SANRA was used to inform the development and reporting of the narrative review. The table provides a descriptive mapping of SANRA criteria to the manuscript and does not represent a formal numerical SANRA assessment or independent quality rating.

Supplementary Table S2. Literature Search Strategy Used to Support the Focused Narrative Review

Database/source	Search date	Coverage	Search purpose	Search concepts and strategy
**PubMed**	August 2026	1 January 2000–30 August 2026	Core temporary-nurse-staffing literature	Temporary staffing terms including agency nurse*, temporary nurse*, travel nurse*, contract nurse*, supplemental nurse*, contingent nurse*, bank nurse*, pool nurse*, float nurse*, nonpermanent/non-permanent nurse*, and per diem nurse* were combined with hospital*, acute care, and inpatient*. Title/abstract field searching was used where appropriate.
**PubMed — Domain 1**	August 2026	1 January 2000–30 August 2026	Nursing shortages and determinants of temporary-staff demand	Core temporary-staffing block AND terms including nursing shortage*, nurse shortage*, workforce shortage*, staffing shortage*, staffing deficit*, staffing gap*, vacanc*, recruitment, retention, labor/labour market, workforce supply, nurse supply, and demand.
**PubMed — Domain 2**	August 2026	1 January 2000–30 August 2026	Workforce flexibility and surge capacity	Core temporary-staffing block AND flexibil*, workforce flexibility, staffing flexibility, surge, surge capacity, elasticity, adaptive capacity, mobil*, redeploy*, demand fluctuation, seasonal demand, service capacity, and staffing capacity.
**PubMed — Domain 3**	August 2026	1 January 2000–30 August 2026	Economic and workforce consequences	Core block AND cost*, econom*, expenditure*, wage*, pay, remuneration, overtime, turnover, retention, vacancy, workload, burnout, job satisfaction, intention/intent to leave, recruitment, workforce stability, and permanent staff/nurses.
**PubMed — Domain 4**	August 2026	1 January 2000–30 August 2026	Clinical integration, orientation, familiarity, and work environment	Core block AND orientation, induction, onboarding, integration, familiar*, situated knowledge, local knowledge, teamwork, collaboration, supervision, work environment, practice environment, unit familiarity, competence/competency, training, education, and support.
**PubMed — Domain 5**	August 2026	1 January 2000–30 August 2026	Patient safety and quality	Core block AND patient safety, quality of care, mortality, failure to rescue, falls, pressure injury/ulcer, infection*, CLABSI, CAUTI, VAP, medication error*, adverse event*, patient outcome*, nursing-sensitive outcome*, length of stay, and readmission*.

**Scopus**	August 2026	1 January 2000–30 August 2026	Cross-database update across the five review domains	Searches used the Scopus TITLE-ABS-KEY field. The temporary-staffing exposure block was combined with TITLE-ABS-KEY(hospital* OR "acute care" OR inpatient*) and the corresponding domain-specific terms. Publication limits were applied using PUBYEAR > 1999 AND PUBYEAR < 2027.
**Scopus**	August 2026	1 January 2000–30 August 2026	Identification of previous evidence syntheses and assessment of conceptual overlap	Temporary-staffing terms were combined with systematic review, scoping review, realist review, narrative review, literature review, integrative review, qualitative synthesis/evidence synthesis, and meta-synthesis/metasynthesis. A broader sensitivity search combined temporary-nursing terminology with review OR synthesis.
**Citation tracking**	Through 30 August 2026	No additional date restriction beyond review scope	Identification of additional primary and review-level evidence	Reference lists and cited literature from key empirical studies and systematic, scoping, integrative, qualitative, realist, and narrative reviews were examined.

Note: The searches were designed to support a focused narrative review rather than an exhaustive systematic review. Search terminology was adapted to database-specific syntax and refined across targeted domain searches. External agency, travel, and contract RNs constituted the primary staffing exposure of interest; studies examining internal bank, pool, float, supplemental, or mixed temporary staffing were retained only when directly relevant. No PRISMA-based systematic selection process or formal study-level risk-of-bias assessment was undertaken. Abbreviations: RN, registered nurse; CLABSI, central line-associated bloodstream infection; CAUTI, catheter-associated urinary tract infection; VAP, ventilator-associated pneumonia; PRISMA, Preferred Reporting Items for Systematic Reviews and Meta-Analyses. In Scopus search syntax, TITLE-ABS-KEY denotes title, abstract, and keyword fields, and PUBYEAR denotes publication year.

Supplementary Table S3. Primary Empirical Studies Informing the Narrative Review of Temporary Nurse Staffing

Study	Setting and design	Temporary-staffing exposure	Main domain	Principal finding	Key interpretive limitation
Aiken et al. [8]	U.S.; cross-sectional secondary analysis of national RN survey and Pennsylvania hospital nurse-survey data	Supplemental/non permanent RNs	Quality/work environment	Temporary nurses had broadly comparable qualifications to permanent nurses; poorer reported quality in hospitals with greater temporary staff use was largely explained by poorer staffing and work environments.	Exposure was broader than external agency nursing; largely cross-sectional and based on nurse-reported outcomes.
Bae et al. [11]	U.S.; unit-level observational secondary analysis of 4,954 RNs in 277 units across 142 hospitals	External temporary RN hours	Dose/safety	More than 15% external temporary RN hours was associated with more reported falls and nurse back injuries; moderate use showed a different pattern for medication errors.	Observational unit-level study; several outcomes were nurse reported; the study-specific threshold should not be interpreted as a universal safe limit.
Hurst and Smith [5]	U.K.; comparative observational study of 605 general and specialist wards	Temporary staff within ward teams	Cost/quality	Wards using temporary staff had greater workload and were more expensive, but overall quality scores did not differ from permanent-only wards.	Temporary staffing was not restricted to external agency RNs; confounding by underlying workload is likely.
Xue et al. [30]	U.S.; retrospective multilevel longitudinal study of 19 units in one tertiary medical center, 2003–2006	Agency-employed supplemental RNs on renewable 3-month contracts	Patient outcomes	Supplemental RN use was not significantly associated with mortality, medication errors, falls, pressure ulcers, or patient satisfaction.	Single institution; unit-level exposure and observational design.

Seo and Spetz [2]	U.S.; longitudinal hospital-level panel/econometric analysis of 326–345 California hospitals per year, 1993–2002	Temporary agency RNs	Demand drivers	Agency demand increased during periods of RN shortage and with inpatient activity, unexpected demand variation, and employee benefit costs.	Historical U.S. labor market; transferability to contemporary health systems may be limited.
Gottlieb and Zenilman [3]	U.S.; national labor-market econometric analysis of travel-nurse job postings, compensation, and geographic mobility during COVID-19 demand shocks	Travel-nurse job postings, compensation, and geographic mobility	Demand/flexibility	Travel-nurse labor supply responded strongly to geographically varying demand and compensation, facilitating redistribution toward high-demand markets; responsiveness was more constrained when demand peaked nationally.	Pandemic-specific labor-market setting; job-market data capture staffing demand and supply responses rather than realized bedside staffing or patient outcomes.
Aiken et al. [9]	U.S.; cross-sectional linked-data observational study of 665 hospitals	Agency-employed supplemental RNs	Mortality/failure to rescue	Agency use was not independently associated with mortality or failure to rescue after accounting for staffing and work environment.	Cross-sectional hospital-level exposure; residual confounding remains possible.
Bae et al. [31]	U.S.; retrospective longitudinal study of 12 ICUs in 6 hospitals, comprising 144 ICU-month observations	Contract/temporary RN hours	Falls/pressure injuries	Higher temporary RN staffing was associated with higher fall and injury-fall rates; pressure ulcers were not associated with temporary staffing.	Small convenience sample and unit-level observational analysis.

Bae et al. [32]	U.S.; retrospective unit-level observational study of 35 acute-care units in 3 hospitals	Temporary nursing staff	Nosocomial infection	Temporary staffing was not significantly associated with CLABSI or VAP; perceived staffing/resource adequacy was associated with infection outcomes.	Limited number of ICUs and aggregated monthly data.
Xue et al. [6]	U.S.; retrospective economic analysis of 19 adult units in one academic medical center	Agency-employed supplemental RNs	Economic outcomes	Modest supplemental RN use was cost-efficient, whereas heavy reliance was not; hourly personnel cost did not differ significantly from overtime by permanent nurses.	Single medical center; findings are dependent on local cost structures.
Bajorek and Guest [24]	U.K.; qualitative case study in A&E departments of two London hospitals	Temporary staff used within emergency-care teams	Permanent workforce	Effects on permanent staff depended strongly on temporary workers' familiarity with the clinical context; unfamiliar staff created additional coordination demands, interruptions, and concerns about service quality.	Small qualitative study; temporary staffing was not restricted exclusively to external agency RNs.
Bethel et al. [27]	U.S.; cross-sectional descriptive survey of 306 travel nurses	Travel nurses	Orientation/integration	Travel nurses identified timely pre-assignment information, day-one system access, unit-specific information, and facility-specific competency assessment as important components of effective onboarding.	Self-selected convenience sample recruited through social media; reported onboarding needs rather than direct patient outcomes.

Dall'Ora et al. [12]	England; retrospective longitudinal observational study of 138,133 patients across 32 medical/surgical units in one hospital	Temporary RN staffing combining hospital-bank and agency staff	Mortality/dose	Modest temporary RN use was not associated with increased mortality, whereas exposure to high levels of temporary RN staffing was associated with higher mortality hazard.	Not agency-specific; most temporary RN shifts were hospital-bank shifts; single hospital.
Ferguson et al. [33]	U.S.; cross-sectional cluster analysis of 605 units in 166 hospitals	Contract/travel nurses	Pressure injuries/falls	Units with the greatest traveler proportion had higher hospital-acquired pressure-injury prevalence/incidence; no consistent relationship was found with falls.	Cross-sectional cluster comparison; residual differences between units may explain part of the association.
Senek et al. [34]	U.K.; cross-sectional survey of 8,841 RNs across acute-care settings	Proportion of temporary nursing staff on reported shifts	Staffing dose/care left undone	Higher proportions of temporary staff were associated with greater odds of reported care left undone, including on otherwise fully staffed shifts; the association increased as the temporary-staff proportion rose.	Cross-sectional self-reported shift data; bank and external agency exposure was not consistently separable; no causal inference.
Polancich et al. [35]	U.S.; retrospective descriptive observational/ecologica l time-series study in one health system during COVID-19	Travel RN FTEs	Length of stay/nursing-sensitive outcomes	Travel RN FTEs correlated positively with average length of stay, while several infection and fall outcomes were not significantly correlated with travel RN staffing.	Small ecological time-series correlations; substantial pandemic confounding.

Gahrmann and Klumb [23]	Switzerland; time-lagged cross-sectional survey of 239 permanently employed hospital nurses	Permanent nurses' perceived exposure to temporary nurses	Permanent workforce	Greater perceived exposure to temporary nurses was associated with lower affective organizational commitment through perceptions of unreasonable and unnecessary tasks.	Self-reported exposure; cross-sectional mediation cannot establish temporal or causal effects.
Spector et al. [25]	U.S.; cross-sectional quantitative survey comparing travel and staff hospital RNs	Travel versus staff RN employment	Workforce outcomes	Travel nurses reported higher turnover intention, physical fatigue, and perceived illegitimate tasks, whereas overall job satisfaction, pay satisfaction, emotional exhaustion, and perceived workload did not differ significantly.	Cross-sectional self-report; relatively small travel-nurse subgroup; findings do not establish that travel employment caused the observed differences.
Griffiths et al. [13]	England; patient-level longitudinal observational cohort of 626,313 admissions across 185 wards in 4 acute hospital trusts	Agency and hospital-bank temporary RNs analysed separately	Mortality/substitution	Low RN staffing increased mortality risk. Temporary staffing partly mitigated the risk associated with low staffing but did not reproduce the staffing configuration associated with adequate substantive RN staffing.	Observational cohort; residual confounding cannot be fully excluded.
Ahmadi Shad et al. [21]	Iran; four-month shift-level time-series analysis of 1,344 shifts across 4 units in one tertiary hospital	Temporary RN deployment	Demand/flexibility	Temporary RNs were more likely to be deployed when permanent RNs were absent, patient numbers were higher, or RN staffing was below average; deployment addressed some but not all staffing shortfalls.	Single hospital and short observation period; temporary staffing was not necessarily external agency-specific.

Fiore-Lopez et al. [26]	U.S.; cross-sectional descriptive survey of 2,201 RNs across 7 healthcare organizations	Travel-nurse employment	Work environment	Travel and nontravel nurses showed no meaningful overall differences in perceptions of the work environment; both groups reported work environments that did not meet healthy-work-environment standards.	Cross-sectional self-report; not a patient-outcome study and not restricted to a single acute-care service type.
Schmelzer et al. [29]	Switzerland; live observational interaction study of 50 nurse huddles involving 309 nurses	Permanent, temporary, and per-diem nursing staff	Communication /integration	Communication patterns differed according to staff consistency; less consistently present per-diem nurses relied more on explicit communication, supporting the relevance of familiarity and shared team mental models.	Communication-process outcome only; observational design; temporary categories were not equivalent to external agency nursing.
Taylor et al. [36]	U.S.; retrospective multicenter linked-database cross-sectional study of 218,789 admissions across 20 PICUs, 2019–2022	Yearly proportion of agency nurses and variability from pre-pandemic baseline	Patient safety	Associations varied by pandemic year and outcome. Higher agency-nurse proportions were associated with VAP in 2020 and with CLABSI and the composite of PICU complications in 2021, whereas associations were not consistent across other years or outcomes.	Pandemic-specific period; annual aggregated staffing exposure and substantial potential for residual temporal and organizational confounding
Saville et al. [22]	England; retrospective longitudinal observational study and economic evaluation of 185 adult acute units in 4 hospital trusts	Permanent versus agency staffing as strategies to eliminate understaffing	Economics/counterfactual	Eliminating understaffing was estimated to be cost-effective, but using permanent staff was more cost-effective and was estimated to save more lives than eliminating understaffing with agency staff.	Economic estimates rely on observational staffing-outcome associations and modelling assumptions.

Pittman et al. [14]	U.S.; multicenter quality-improvement study using 2019–2022 data from 70 hospitals	Agency nurse hours	Patient safety/dose	Agency hours were associated with selected outcomes, particularly pressure ulcers and perioperative hemorrhage/hematoma, but adverse associations were not universal across the 10 indicators examined.	Observational/quality-improvement design; mostly pandemic-era data; hospital heterogeneity.
Bartock et al. [15]	U.S.; single-center retrospective observational analysis of staffing and quality data, 2019–2023	Agency/travel RN shifts	Patient safety/infections	Agency staffing above 10% was independently associated with higher total PSI burden; after adjustment, infection outcomes were explained more strongly by case mix than by agency-staffing tier.	Single-center observational study; strong temporal/pandemic confounding; no causal inference.

Abbreviations: RN, registered nurse; A&E, accident and emergency; ICU, intensive care unit; FTE, full-time equivalent; CLABSI, central line-associated bloodstream infection; VAP, ventilator-associated pneumonia; PICU, pediatric intensive care unit; PSI, patient safety indicator; U.S., United States; U.K., United Kingdom. Note: Temporary nurse staffing is used as an overarching term. External agency, travel, and contract RNs constituted the primary exposure of interest. Evidence concerning hospital-bank, pool, float, supplemental, or mixed temporary staffing is identified explicitly and should not be interpreted as evidence specific to external agency nurses. Study-specific staffing thresholds or breakpoints are not universal limits for appropriate or safe temporary staff use.
